# Posttraumatic stress disorder among female street-based sex workers in the greater Sydney area, Australia

**DOI:** 10.1186/1471-244X-6-24

**Published:** 2006-05-24

**Authors:** Amanda Roxburgh, Louisa Degenhardt, Jan Copeland

**Affiliations:** 1 National Drug and Alcohol Research Centre, University of New South Wales, Sydney, Australia

## Abstract

**Background:**

This paper examines rates of exposure to work-related violence and other trauma, and the prevalence of lifetime and current posttraumatic stress disorder (PTSD) among female street-based sex workers. It also investigates associations between current PTSD symptoms and: demographic characteristics, psychiatric comorbidity, injecting and sex risk behaviours, and trauma history.

**Methods:**

Cross sectional data collected from 72 women via face to face structured interviews. The interview included structured diagnostic assessment of DSM-IV PTSD; drug dependence; depression; experience of childhood trauma; and an assessment of sex working history.

**Results:**

All but one of the women interviewed reported experiencing trauma, with the majority reporting multiple traumas that typically began in early childhood. Child sexual abuse, adult sexual assault and work related violence were commonly reported. Just under half of the women met DSM-IV criteria for PTSD and approximately one-third reported current PTSD symptoms. Adult sexual assault was associated with current PTSD symptoms. Depression and drug dependence were also highly prevalent; cocaine dependence in particular was associated with elevated rates of injecting risk and sexual risk behaviours.

**Conclusion:**

These women reported complex trauma histories and despite ongoing opportunities for clinical intervention, they continued to experience problems, suggesting that current models of treatment may not be appropriate. More targeted interventions, and integrated mental health and drug treatment services are needed to address the problems these women are experiencing. Outreach services to these women remain a priority. Education strategies to reduce risky injecting and sexual behaviours among sex workers should also remain a priority.

## Background

There is a long history of women engaging in the sex industry, both in developed and developing countries, and a large body of literature exists on the risks these women face in the course of their work [1]. Previous research has documented the risks of blood borne virus (BBV) transmission and sexually transmitted infections among sex workers due to unprotected sex with clients [2], the relatively high rates of HIV among sex workers in some countries, and the potential risks posed to the broader community via BBV transmission through clients to the general population [3].

There is good evidence to suggest that sex workers are also highly likely to encounter violence during the course of their work [4-6]. In a study of females who conducted sex work indoors compared with women working outdoors, the outdoor sex workers were significantly more likely to report ever having experienced work-related violence (81% compared to the indoor sex workers (48%) [7]. In addition, street-based sex workers in particular may face risks of work-related violence due to the locations where they provide services, and the nature of the interaction with their clients [6]. Kurtz et al [6] examined the characteristics of female sex workers who had recently (in the past month) been victimised compared to those who had not and found that women who were homeless, had used crack, and injected any drug in the past month were more likely to report recent victimisation than women who were not homeless, and had not used crack or injected any drug recently. Having sex, or even getting in the car with a client was also significantly associated with recent victimisation. Controlling the location and destination of services provided was a key factor in these women's safety.

Exposure to traumatic events during the course of occupational duties is associated with psychological problems, one of which is posttraumatic stress disorder (PTSD) [8]. Previous research investigating the prevalence of PTSD in certain occupational groups has suggested that rates among those exposed to traumatic events are typically greater than those reported in the general population. These groups include police officers (current PTSD prevalence up to 9%) [9] combat veterans of the Vietnam and Gulf Wars (current PTSD prevalence up to 15%) [10-12], and journalists in war zones (lifetime rates of 29%) [13]. In comparison, the 12 month prevalence of PTSD in the Australian population has been estimated at 3.3% [14].

Given the risk of exposure to traumatic events during the course of their work, this study focuses on violence and trauma as an occupational risk among street-based sex workers, and the psychological problems that may be associated with such experiences. Despite the stigma surrounding street-based sex work, it is a legal occupation in New South Wales (NSW). The legislation states that sex workers may operate along public thoroughfares as long as they are not within view of a dwelling, church, school or hospital. NSW is unique in this respect as no other state or jurisdiction in Australia permits street-based sex work [15].

### Posttraumatic stress disorder

The diagnosis of PTSD describes symptoms that develop in response to exposure to "extreme traumatic stressors involving direct personal experience of an event...or witnessing an event" [16]. These events include natural disasters, witnessing serious injury or death, serious accidents, exposure to combat, child sexual abuse, child neglect, physical assault, child physical abuse, being threatened with a weapon, tortured or held captive, and rape. Symptoms range from re-experiencing the trauma, persistent avoidance of reminders of the event, numbing of responsiveness, and persistent anxiety or hyper-arousal. For a diagnosis of PTSD, these symptoms must be present for more than one month, and must cause clinically significant distress or impairment in functioning [16].

Not all exposure to trauma results in a diagnosis of PTSD [17], but several factors have been associated with an increased risk of developing PTSD following trauma exposure. These include background variables such as childhood trauma, comorbid mental health problems, family instability and substance abuse [18-21]. There is also good evidence to suggest that females are at greater risk than males of developing PTSD following trauma [22,23].

Characteristics of the trauma also affect the likelihood of development of the disorder: PTSD is more likely to develop in response to rape [24], and associated symptoms are more likely to be severe and persistent following an event of human design (e.g. rape and torture) [16]. Continued exposure to trauma is another risk factor for development of PTSD, with previous research suggesting that the longer the exposure, the more persistent and/or severe PTSD symptoms will be. These findings relate to war veterans [10,25], as well as individuals who have experienced child sexual abuse [8]. If this relationship holds for street-based sex workers, one would assume that the longer they are exposed to traumatic experiences in their workplace, the more persistent their PTSD is likely to be.

Previous literature suggests that sex workers may have many of these risk factors. Experiences of childhood trauma are commonly reported among sex workers [26], and experiences of adult sexual assault [27] and violence while working [5,28-30] are prevalent. Adult sexual assault has also been associated with psychiatric morbidity among street-based sex workers [31].

A comparative study conducted in New Zealand [32] found that sex workers were significantly more likely to report adult sexual assault (55%) than non-sex workers (13%). Likewise, Surratt et al [33] found that half of the female street-based sex workers they interviewed reported child sexual abuse and 40% had experienced work-related violence in the previous twelve months.

The literature also makes reference to the connection between childhood violence and later re-victimisation. Surratt et al [33] purported that consistent relationships between historical and current victimisation among female street-based sex workers suggested a continuing cycle of violence in these women's lives, and that they operate within a 'subculture of violence'. Tyler at al [26] found that among a group of homeless females, those with a history of sexual abuse were more likely to be re-victimised on the street. Re-victimisation among this group was also associated with trading sex for money, while substance abuse was associated with sexual victimisation.

Research indicates that mental health problems are also prevalent among sex workers [34]. One comparative study in Scotland examining differences in psychiatric morbidity between female drug users who engaged in sex work versus those who did not [35] found that sex workers were significantly more likely to report adult physical and child sexual abuse, to have attempted suicide and to meet criteria for current depressive ideas than non sex workers. Similarly, research in the United States found that sex workers exhibited significantly higher levels of psychological distress, independently of having experienced traumatic events [31].

High rates of family instability have also been reported. In a comparative study of female sex workers and females who had experienced child sexual abuse, sex workers reported experiencing higher rates of parental separation and less parental care [36]. Child sexual abuse has also been linked with family dysfunction, leaving home at an earlier age, living on the streets for longer periods of time and engaging in sex work [26].

The literature on PTSD also suggests that the diagnosis is associated with other psychological problems [37]. Research on occupational groups at high risk of PTSD (e.g. war veterans, journalists in war zones, and police) has found that PTSD symptomatology is significantly associated with alcohol [13,38] and other substance use [39]. Research among police officers in the U.S. suggests that comorbid PTSD and problematic alcohol use is associated with increased risk of suicidal ideation [38]. Comorbid substance use is also likely to complicate treatment for PTSD [40].

There is good evidence to suggest that rates of drug use among street-based sex workers may be higher than in the general community. Studies have found high rates of illicit drug use [41], injecting drug use [2,5,42,43], and drug dependence [2,35,44] in a number of countries. Studies report that between 57% and 90% of street-based sex workers report injecting drug use, and between 46% and 96% report drug dependence [34,35,44]. Problematic substance use is also likely to complicate PTSD and response to treatment among street-based sex workers.

Given the high rates of childhood trauma, family instability, mental health problems and problematic substance use among street-based sex workers, they may be at high risk of developing PTSD if they are exposed to traumatic events. In support of this, one study reported that 68% of female sex workers interviewed met criteria for lifetime diagnosis of PTSD [4]. This was associated with exposure to trauma in childhood and adulthood, as well as high levels of work-related violence. In addition, the more types of violence reported (childhood physical and sexual abuse, rape and physical assault while working), the greater the severity of PTSD symptoms [4]. In another study of sex workers in Israel [45] 17% of the women reported having experienced PTSD symptoms in the past month. Child sexual abuse (33%), parental neglect as children (30%), rape (30%) and physical assault (30%) while working were prevalent among these women. Consistent with Farley and Barkan's [4] findings, symptoms of PTSD among these women were positively associated with past and work-related traumas.

### Aims of the current study

There has been no Australian research on PTSD or its association with mental health, drug use and risk behaviours among street-based sex workers. Investigation of these issues may provide important information for the development of targeted interventions for this group. The rationale for examining street sex workers in the current study is empirically based, with previous studies suggesting that they are a more marginalised group than non street sex workers, being more vulnerable to adverse contact with law enforcement, subject to physical assault, rape, kidnap, and being threatened with a weapon [5,28-30].

The aims of the current study were therefore:

1. To examine demographics, sex work history, working conditions and work-related risk behaviours;

2. To examine rates of exposure to work-related violence and other traumatic events;

3. To examine the prevalence of posttraumatic stress disorder (PTSD) and current PTSD symptoms;

4. To investigate associations between current PTSD symptoms and a range of other issues such as psychiatric comorbidity and risk behaviours; and

5. To examine other characteristics (such as mental health and drug use) that may impact on current PTSD symptoms.

## Methods

This study collected cross-sectional data between April and August 2005 via a structured interview administered face-to-face. Seventy two participants were recruited through various agencies that have ongoing contact with female street-based sex workers through the provision of on-site and outreach services. Recruitment cards with a contact number were distributed by these agencies to potential participants, who then called to organise an interview time. Participants were 17 years and over and currently engaged in street-based sex work. In order to maintain participant confidentiality, no identifying details were recorded on the questionnaire, only a study number. Written consent was obtained from all participants. This project was approved by the University of New South Wales (HREC 04277), Sydney South West Area Health Service (05/018) and South Eastern Sydney Area Health Service (04/334) Research Ethics committees.

The questionnaire collected information on demographics, working conditions in the sex industry, drug use and dependence, injection-related risk behaviour, suicidal ideation, depression, trauma history (including child sexual abuse, adult sexual assault, and violence at work) and PTSD. Trauma histories and diagnoses of PTSD were obtained using the National Mental Health and Well-Being (NHMWB) version of the Composite International Diagnostic Interview (CIDI). The CIDI is a fully structured diagnostic interview for the assessment of mental disorders. It provides diagnoses in accordance with two major psychiatric classification systems, the International Classification of Diseases, 10^th ^Edition (ICD10) and the Diagnostic and Statistical Manual of Mental Disorders, 4^th ^Edition (DSM-IV) [14,46,47]. Criteria for a lifetime diagnosis of PTSD were determined in accordance with DSM-IV criteria [47]. Previous research has established that the CIDI has good psychometric properties [48]. The Beck Depression Inventory-II (BDI-II) [49] was administered to determine the presence and severity of current depressive symptoms. The BDI-II is a 21 item self-reported measure, designed to represent the criteria for a major depressive episode as presented in the DSM-IV [16]. Scores on the BDI-II range from 0 to 63, with scores between 0 and 13 representing minimal depression, 14 to 19 representing mild depression, 20 to 28 representing moderate, and 29 to 63, severe depression. Dependence on various drugs (cocaine, heroin and cannabis) was assessed using the Severity of Dependence Scale (SDS), a five-item 15-point scale that measures psychological dependence on various illicit drugs [50]. The injecting sub-scale of the HIV Risk Taking Behaviour Scale, a component of the Opiate Treatment Index (OTI), was used to measure current injection-related risk behaviour [51]. Data on child sexual abuse and adult sexual assault was collected utilising a structured instrument from a child development study conducted in Christchurch, New Zealand [52].

Three researchers, experienced in interviewing individuals engaging in illegal activities, were trained to administer the interview (including structured scales such as the CIDI and the SDS). With the exception of the BDI-II (which is self-report) the questionnaire was administered by the interviewers. If participants were having difficulty reading the BDI-II this was administered by the interviewers. More detail on the questionnaire is available elsewhere [53].

### Statistical analyses

Descriptive statistics were used to record the prevalence of PTSD, depressive symptoms and drug dependence. Odds ratios and chi square analyses were conducted to determine the relationship between PTSD and other variables. Independent samples T tests were used to look at differences in the mean age of those women with current PTSD and those without, while the Mann-Whitney U statistic was used to examine differences in the median number of traumas between these groups. Post-hoc tests were conducted looking at the differences between women who identified as being of Aboriginal and/or Torres Strait Islander (A&TSI) origin and those who did not on; homelessness in the past 12 months (Chi square); level of depression; and age of entry into sex work (t-tests). Multiple logistic regression, with the backwards elimination method using log likelihood ratios, was employed to model associations between current PTSD and other variables at a multivariate level. All analyses were conducted using SPSS for windows, version 12.0 (SPSS Inc, 2003).

## Results

### Demographic characteristics

Demographic characteristics of the sample are presented in Table 1. The mean age of the sample was 34 (SD 8.8, range 18 to 58). Approximately one quarter (23%) of the sample identified as being of Aboriginal and/or Torres Strait Islander (A&TSI) origin. The mean years of school education was 9, with only 18% of the sample attaining a high school education. Almost two-thirds (61%) of the sample reported moving out of home before age 16. The mean age of living independently was 15, and this was also the most common age of living independently (range 11 to 21 years). A substantial minority (14%) reported having no fixed address, or current homelessness and nearly half (45%) of the sample reported being homeless within the past 12 months. A larger proportion (68%) of women who identified as being of A&TSI origin reported being homeless in the past 12 months compared to women of non A&TSI origin (38%). This finding was close to statistical significance (p = .05).

**Table 1:** Demographic characteristics of the sample

**Characteristic**	% (N = 72)**
Mean age in years	34
Mean years of school education	9
Completed secondary education	18 (13)
Aboriginal and/or Torres Strait Islander (A&TSI)	23 (16)
Prison history	56 (40)
Currently homeless	14 (10)
Homeless in past 12 months	45 (32)
Currently in drug treatment	61 (44)
Source of income in past month (apart from sex work)*	
wage or salary	6 (4)
government pension	89 (64)
criminal activity	17 (12)
child support	6 (4)
Sex work as main source of income in past month	93 (67)
Left home before 16 years of age	61 (44)

*Percentages do not add to 100 as more than one response was possible.

**The figure in brackets represents the N that the percentage refers to.

Very few of the participants (6%) reported receiving income from a wage or salary from other paid work and most (89%) cited the government pension as an alternative source of income. Likewise, most of the sample (93%) reported sex work as their main source of income in the past month (Table 1).

### Working environment

The mean age at which participants reported starting sex work was 21 (range 12 to 55 years), with nearly one-third of the sample (31%) starting before the age of 18 years (Table 2). There were no differences in the mean age of commencing sex work among the A&TSI women (21 years of age) and the non-A&TSI women (22 years of age). The mean length of involvement in the sex industry was 12 years (SD 7.3, range four months to 39 years). Three quarters of the women (75%) reported providing services on the street and 67% reported providing them in cars. Two-thirds (66%) of the sample reported that they found sex work very stressful. The majority of women (85%) reported having experienced violence while working, however only 35% had reported these incidents to police. The most common work-related incidents reported were physical assault (65%), rape with a weapon (40%), and rape without a weapon (33%) (Table 2).

**Table 2:** Sex work history and working conditions.

**Variable**	% (N = 72)*
Mean age (years) started sex work	21
Commenced sex work before age 18	31 (22)
Mean number of years involved in sex work	12
Location where services provided	
street	75 (54)
cars	67 (48)
safe house	57 (51)
Always use condoms when have sex with clients	82 (59)
Always use condoms during oral sex with clients	57 (41)
Find sex work very stressful	66 (48)
Aspect that is stressful	
clients	50 (36)
violence	11 (8)
lack of work	11 (8)
self esteem issues	11 (8)
long hours	10 (7)
Ever experienced violence while working	85 (61)
Types of work-related violence	
physical assault	65
rape at gun/knife point	40
rape without a weapon	33
robbery	29
attempted rape	21
threatened/attacked with a weapon	17
abduction	13
Ever reported these incidents to police	35 (25)

*The figure in brackets represents the N that the percentage refers to

### Drug use history

The vast majority (94%) of the sample had ever injected any drug (Table 3). The majority (83%) had injected heroin in the past month on a median of 30 days, just under half (42%) reported injecting cocaine in the past month on a median of 17.5 days, and nearly two thirds (63%) reported cannabis use in the past month on a median of 20 days. The majority (82%) of the sample was heroin dependent according to the Severity of Dependence Scale (SDS), and approximately one-third were cocaine (36%) and cannabis (32%) dependent (Table 3).

**Table 3:** Drug use history, dependence and relationship to sex work.

**Variable**	**% (N = 72)**
Ever injected any drug	94 (68)
Mean age in years first injecting drug use	19
Injected before age 16	23 (16)
Heroin dependent	82 (59)
Cocaine dependent	36 (26)
Cannabis dependent	32 (23)
Borrowed used needles in past month	7 (5)
Lent used needles in past month	22 (16)
Shared other injecting equipment in past month	32 (23)
Commenced injecting drug use prior to sex work	53 (38)
Commenced sex work prior to injecting drug use	26 (18)
Reporting drug use had increased since starting sex work	71 (51)

*The figure in brackets represents the N that the percentage refers to

Approximately one-third (32%) of the sample reported sharing injecting equipment such as spoons, water, filters and tourniquets in the past month (Table 3). Those women who were cocaine dependent were 4.6 times more likely to have shared injecting equipment in the past month (χ^2 ^= 6.85, CI 95% 1.6 to 13.7) than those who were not. They were also less likely than those who were not cocaine dependent to use condoms when having penetrative sex with clients (χ^2 ^= 4.19, OR .20, CI 95% .04 to .81).

When asked about the relationship between drug use and their involvement in the sex industry, just over half of the women (53%) reported that they used drugs to facilitate their sex work. The mechanism of drug use to facilitate sex work was generally described as 'numbing' so that the women 'did not have to think' about what they were doing, and they didn't 'have to feel' while working. Just over half of the women (52%) reported engaging in sex work to pay for drugs, with 7% reporting that drugs are the reason they work. Eighteen percent of the sample reported the relationship as being reciprocal (i.e. they used drugs to facilitate sex work and worked to facilitate drug use).

### Depression and suicidal ideation

The majority of the sample (87%) reported the presence of some depressive symptoms ranging between mild and severe, while more than half (54%) reported severe current depressive symptoms in accordance with the Beck Depression Inventory (BDI-II). Approximately three quarters (74%) of the sample reported having ever thought about suicide, and just under half (42%) reported having tried to kill themselves. Just under half (40%) of the sample reported speaking with a health professional about a mental health problem other than their drug use in the past six months. Among those who had consulted a mental health professional recently, depression (79%) was the most common reason for this consultation. A&TSI women reported significantly higher levels of depression (BDI mean of 36.5) compared to non-A&TSI women (27.6) (t = 2.8, df = 68, p = .007). Very few of the A&TSI women (25%) had spoken to a mental health professional in the past six months compared with the non-A&TSI women (45%).

### Trauma

All but one of the participants (99% of the sample) reported having experienced at least one traumatic event in their lifetime, with a large proportion (93%) reporting multiple traumas. More than half (53%) of the sample reported experiencing 6 or more traumatic events. Three quarters (75%) of the sample reported experiencing some form of sexual abuse before the age of 16 years, and the mean age of first occurrence was 7 years (range 1 to 15 years). Approximately one quarter (26%) of the sample reported that the first incident occurred before the age of 6 years. Approximately half (51%) of the sample reported that someone had vaginal sex with them before they were 16 years. The majority of the sample (81%) reported having been raped while working or in their personal lives (44% of the sample reported being raped outside of work) and physically assaulted (81%), while 71% had witnessed someone being badly injured or killed. Among those exposed to trauma, the largest proportions reported rape (19%) and being threatened with a weapon or being held captive (19%) as the most stressful of the traumatic events they had experienced (Table 4).

**Table 4:** Prevalence of exposure to traumatic events and events perceived as most stressful.

Trauma***	% (N = 72)**	% most stressful event*
Child sexual abuse (CSA)		
ever experienced CSA	75 (54)	13 (9)
mean age of first CSA	7	
reporting first CSA before age 6 years	26 (19)	
reporting someone having vaginal sex with them before age 16 years	51 (37)	
Rape	81 (58)	19 (13)
Physical assault	81 (58)	12 (8)
Witness serious injury or death	71 (51)	15 (10)
Threatened with a weapon/held captive	68 (49)	19 (13)
Child physical abuse	54 (39)	12 (8)
Life threatening accident	51 (37)	4 (3)
Child neglect	38 (27)	4 (3)
Natural disaster	26 (19)	1 (1)

* Among those who had experienced a stressful event.

**The figure in brackets represents the N that the percentage refers to.

***With the exception of some of the child sexual abuse items all trauma data presented in this table derives from the PTSD scale in the CIDI

### Posttraumatic stress disorder

Almost half (47%) of the sample met DSM-IV criteria for a lifetime diagnosis of PTSD. For 91% of those with PTSD, their symptoms were chronic in duration (i.e. they lasted for 3 months or longer), and 82% reported that their symptoms lasted for one year or more.

Among those with PTSD, a median of 17 years (range 1 to 52) had passed since the most stressful traumatic event occurred. Despite this, 62% of those who met criteria for PTSD (31% of the sample) met DSM-IV criteria for current PTSD (i.e. within the preceding 12 months). Approximately three quarters (74%) of those participants who developed PTSD said they had spoken to a health professional about the associated symptoms.

Table 5 sets out a comparison of those women who reported current PTSD symptoms with those who did not on a range of variables. There were no differences between the groups in demographic characteristics. Similarly, there were no differences in age of initiation of injecting drug use, drug dependence, or injecting risk behaviours. Age of entry into sex work, and sex risk behaviours were also similar for both groups.

**Table 5:** Correlates of current PTSD symptoms.

**Variable**	Current PTSD % (N = 22)**	**No current PTSD (N = 50)**
**Demographics**		
Mean age in years	34	33
Homeless in the past 12 months	50 (11)	42 (21)
Median years of school education	9	9
A&TSI status	27 (6)	20 (10)
**Drug use**		
Median age in years first injecting drug use	17	18
Drug dependent		
heroin dependent	73 (16)	86 (43)
cocaine dependent	32 (7)	38 (19)
cannabis dependent	36 (8)	30 (15)
Shared injecting equipment in the past month	20 (4)	40 (19)
**Sex work & sex risk behaviours**		
Median age started sex work	20	18
Always use condoms when have sex with clients	91 (20)	83 (39)
Always use condoms during oral sex with clients	62 (13)	60 (28)
**Mental health & trauma**		
Median number of traumas experienced	7**	5
Severe depressive symptoms	73^+^(16)	48 (23)
Attempted suicide	50 (11)	40 (19)
Experienced physical assault while working	77 (17)	77 (39)
Ever experienced child sexual abuse	82 (18)	72 (36)
Ever experienced child neglect	59* (13)	28 (14)
Ever experienced adult sexual assault	82* (18)	53 (26)
Median age of first sexual assault	13	14

* p < .05 ** p < .01 +close to significance p = .05.

**The figure in brackets represents the N that the percentage refers to

There were differences in trauma histories between the two groups. Women reporting current PTSD were nearly 4 times more likely to have ever experienced adult sexual assault than women who did not report current PTSD (82% vs. 53% respectively; χ^2 ^= 4.18, OR 3.98, 95% CI 1.2 to 13.5), and they had also experienced a significantly greater number of traumas (median of 7 traumas) than those without current PTSD (median of 5 traumas) (Mann Whitney U = 329, df = 70, p < 0.01). In addition, women reporting current PTSD were nearly 4 times more likely to report being seriously neglected as a child (59%) than women without current PTSD (28%) (χ^2 ^= 5.04, OR 3.7, 95% CI 1.2–10.6). There were no differences in proportions reporting child sexual abuse (82% among those with current PTSD and 72% among those without current PTSD), or physical assault at work (77% each). Likewise, there was no difference between the groups in median age of first sexual assault (13 for those with current PTSD, and 14 those without current PTSD). The association between current PTSD and severe depressive symptoms was close to significance, with women reporting current PTSD more likely to report being depressed at the time of interview (p = .05). Variables that were significant at the bivariate level (number of traumas experienced, severe depression, child neglect and adult sexual assault) were then entered into a multiple logistic regression model. The only variable that remained significant was the number of traumas experienced (Wald statistic = 6.87, p = .009, OR 1.49, CI 95% 1.11 to 2.00). Women reporting current PTSD were more likely to have experienced a greater number of traumas than those without current PTSD

## Discussion

This study examined rates of exposure to work-related violence and other traumatic events, and the prevalence of lifetime and current PTSD among female street-based sex workers. It also investigated associations between current PTSD and demographic characteristics, psychiatric comorbidity, injecting and sex risk behaviours and trauma history.

The overwhelming majority of women interviewed for this study reported multiple traumas in their lifetime, with over half experiencing 6 or more events. These women had many of the markers reported in the literature (childhood trauma, family instability, mental health problems, rape and substance use) as being associated with the risk of developing PTSD following exposure to traumatic events. The majority had experienced child sexual abuse before the age of 16 years, a substantial proportion reported being seriously neglected as a child, and over half of the women reported leaving home before age 16 years. The majority of women had experienced adult sexual assault, and drug dependence, severe depressive symptoms and suicidal ideation were prevalent. These findings are entirely consistent with previous research among sex workers [4,5,27,31,54]. Just under half of the sample reported being homeless in the previous twelve months, and homelessness was particularly prevalent among the A&TSI women. In addition, A&TSI women reported significantly higher levels of depression compared to non-A&TSI women, with very few of these women having spoken to a mental health professional in the past six months.

Just under half of the women met criteria for lifetime PTSD, and approximately one-third reported current PTSD symptoms (31%), a rate that is almost ten times higher than that in the general population (3.3%) in Australia [14], and higher than the upper levels of prevalence rates among other occupational groups (war veterans 15%; police officers 9%) [10,38].

Those women meeting criteria for current PTSD were more likely to report a greater number of traumas, serious neglect during childhood, and adult sexual assault. This last point is particularly important, as these women continue to be exposed to the risk of sexual assault through their work, the very factor that is associated with their current PTSD symptoms. These women are at ongoing risk of further work-related trauma, so whilst they remain in the street-based sex industry, their PTSD symptoms are unlikely to recede.

### Clinical implications

Although the majority of women who met criteria for a lifetime diagnosis for PTSD reported having consulted a professional about issues associated with their trauma, a substantial proportion continued to experience PTSD symptoms. Likewise, despite almost half the women reporting consulting a mental health professional in the past 6 months, high proportions reported severe current depression. It is important to consider then, whether traditional mental health care services are appropriate for this group, who have complex histories and high levels of psychiatric morbidity. Mental health professionals need to be aware of the issues that are central for this group, particularly with respect to child sexual abuse and ongoing sexual assault, which often engenders a lack of trust and difficulty with disclosure. There are also issues of stigma surrounding sex work that may prevent these women from engaging in therapy, and these may need to be addressed. Very few indigenous women in this study reported *any *engagement with mental health services, and strategies to encourage this group to access such services are clearly required. Employing A&TSI mental health professionals in key areas may assist with these objectives.

There are several factors that complicate treatment of PTSD among these women, one of which is the high prevalence of drug dependence. Central to conventional cognitive behavioural approaches to PTSD intervention is the ability to cognitively confront traumas experienced, and assault victims who develop PTSD are characterised by extreme cognitive and behavioural avoidance [24]. There was some evidence to suggest that drug use among the women in this study was serving the purpose of reducing psychological distress through cognitive avoidance. This avoidance will undoubtedly affect treatment, and any psychological intervention for PTSD among these women should ensure that drug use is addressed. Treatment is further complicated by the interplay between PTSD and substance use, with previous research showing that unremitted PTSD is associated with poorer outcomes for substance use disorders [55].

Drug use is also important to target in order to reduce some of the associated risks. Research among police officers suggests that comorbid PTSD and substance use is associated with higher risk of suicide [38], and the high prevalence of suicide attempts among this group (42%) suggests this risk may be elevated among street-based sex workers. Previous research confirms that sex workers with a history of child sexual abuse and adult sexual assault are at elevated risk of attempting suicide compared to non-sex workers with a similar history [35], and these histories are prevalent among the women in the current study. In addition to the risk of suicide, cocaine dependence was associated with increasingly risky injecting and sexual behaviours. Education strategies for safe sex and drug use then clearly need to target these higher-risk injectors. More practically, targeting drug use may reduce the financial pressures for high-risk sex workers.

Another factor complicating treatment is the ongoing exposure to work-related trauma for these women. Much of the research on successful PTSD intervention recommends removing clients from the potential of exposure to further trauma [24] and establishing a safe environment before commencing therapy [20]. Given that current PTSD among these women is related to adult sexual assault (which was reported as the most prevalent and most stressful trauma), establishing a safe environment and minimising their ongoing exposure to trauma would entail leaving the sex industry, where occupationally they are at risk of sexual assault on a daily basis. This may be difficult for these women as many of them reported low levels of education. Sex work was the main source of income for the vast majority of women, suggesting that they have limited employment alternatives. Indeed, some of the women cited limited alternatives and lack of other job skills as reasons for remaining in the sex industry.

The nature of PTSD interventions may also be difficult to employ with these women. Imaginal exposure (in which the client relives the trauma in their imagination with the assistance of the therapist) is a central component of therapy [56], and has received strong support as an effective treatment for PTSD among sexual assault survivors [24]. It is difficult to implement when there is ongoing trauma, as it is a psychologically demanding strategy [40]. In addition, social support is an important protective factor in minimising these demands, but very few of these women reported supportive relationships. Imaginal exposure would not be an appropriate strategy to employ with these women until such time as their physical safety could be guaranteed.

Conventional PTSD interventions may not be effective for these women, and alternative strategies may need to be employed. Given the issues of cognitive avoidance and drug use among this group, more behaviourally focused strategies may be useful. These might include harm reduction strategies such as teaching the women how to recognise the signs of distress, and how to minimise them. There is some evidence to suggest that simple relaxation techniques may be successful in minimising trauma-related distress among sexual assault victims [57] and they require relatively few cognitive demands [58].

Increased awareness of and access to crisis telephone lines and mental health services may also be useful. Agencies providing outreach services to this group could promote and provide mental health and referral contacts to those women wishing to seek assistance. Provision of mental health services via outreach would also be a useful adjunct to existing services. Research in the U.S. suggests that the provision of brief psychological interventions for female street-based sex workers via existing outreach services is effective in reducing the frequency of drug use [59], and increasing drug treatment uptake and retention [60]. Motivational interviewing (a method designed to evoke intrinsic motivation for change in health risk behaviours by resolving ambivalence [61] has been shown to be particularly useful for targeting drug use among street-based sex workers [59]. While there is evidence against the efficacy of brief interventions targeting PTSD symptoms [62], these techniques may be useful for targeting other mental health problems among this group, thereby reducing the associated risks.

Psychological interventions for street-based sex workers should be specifically tailored to their needs, and they should be flexible, as many of these women have little stability in their lives. High levels of homelessness make it difficult for these women to access community resources, as well as complicating agency service provision. Accordingly, assistance with welfare and access to housing should remain a priority for agencies that serve as a first point of contact for this group, as without such stability drug treatment programs and psychological interventions are unlikely to be effective.

At a more basic level, the ongoing risk of exposure to trauma among these women needs to be targeted, as it is associated with current PTSD in this group. There needs to be continued liaison between the police and outreach workers about the negotiation of legal and safe places for these women to work. A high proportion of women in the current sample reported providing their services in cars, which may increase the risk of work-related violence. The overwhelming majority reported experiencing work-related violence however, very low proportions had reported these incidents to police. In order to encourage more women to report work-related violence, there should be an ongoing police commitment to the provision of sex work liaison officers in the local areas where street sex work is conducted. Many of the women reported positive experiences with liaison officers when reporting assaults, and any encouragement for these women to engage with police is important as it may reduce the risks they face on a regular basis. Conflict between police and street-based sex workers most often occurs due to the women soliciting in an area that is prohibited under the Summary Offences Act 1988, 19(1). Police are under constant pressure from local businesses and residents to move the women out of these areas, which can result in the police pressuring the women. Ongoing dialogue between police, outreach workers and sex workers would not only help to relieve some of this tension, but it would also minimise the disruption to the broader community [5]. Finally, safety measures such as the provision of personal distress alarms through outreach services may minimise the risk of repeated exposure to trauma for these women.

### Future research

These findings have several implications for further research. Firstly, the issue of safe houses needs to be addressed, as a proportion of women in the current sample continued to provide services in cars, despite safe houses being available in the Kings Cross area. Research is needed to investigate the reasons safe houses are not being utilised, as well as what changes might be required to encourage greater use of existing safe houses. This research may provide vital information for the establishment of additional safe houses outside the inner city area.

Given the prevalence of cocaine dependence among this group and the increased potential for injection-related and sexual risk behaviours, further research is required on if, and how, sex workers use drugs to facilitate their work, and what impact, if any, this has on the nature of the encounter (e.g. Does it limit their ability to negotiate safe sex? Does it increase their vulnerability to violence?).

Future research among street-based sex workers should also consider the impact that A&TSI status may have for these women, as our findings suggested that women identifying as being of A&TSI origin were more likely to report depressive symptoms and less likely to access mental health services.

Finally, more research is required on the nature of psychological treatment that would be most effective for street-based sex workers, and trialling brief psychological interventions via existing outreach services would be a useful start.

### Limitations

The findings of this study refer to street-based sex workers, who differ from sex workers employed in other sectors of the industry on several domains [5,28,34]. Street-based sex workers in other Australian jurisdictions are also likely to differ from the current sample due to different legislation.

Inherent to any study of marginalised populations engaging in stigmatised activities is the issue of sample representativeness, which is difficult to achieve among these groups. Findings from the current study may be more indicative of those women who were willing to participate. One recruitment strategy utilised in an attempt to overcome this limitation was personal introductions to the women, facilitated by outreach workers who knew the women and the areas well.

## Conclusion

The female street-based sex workers interviewed for this study reported complex histories of trauma, and the majority reported experiencing work-related violence. Current PTSD was more prevalent among these women than in the general population, and may be complicated by ongoing exposure to trauma, due to risks they face every day at work. These findings raise several issues. Firstly, outreach services to street-based sex workers remain crucial, in order to provide links with health and welfare services, and strategies to increase these women's personal safety. Second, that so many of the women continue to experience mental health problems, despite access to health services suggests that current models of service provision are not sufficient to address the problems among this group. More targeted intervention programs and more integrated models of care need to be developed given the high comorbidity of mental health and substance use problems, and the interplay between the two. Despite the legality of street-based sex work in NSW, it is an occupation that continues to be surrounded by stigma, which impacts on these women reporting work-related violence. The legality of street-based sex work in NSW does not provide sufficient protection against this violence and again, ongoing outreach efforts of harm minimisation, including police liaison, are crucial. Every effort should be made to encourage these women to report incidents to the police in an effort to minimise the ongoing risks they face at work. Finally, while education that targets safe sex and injecting practices among sex workers should remain a priority (given the high rate of problems encountered among this group, and the risks they face due to contact with multiple sex partners), it is recognised that these strategies will remain compromised within the context of the high prevalence of rape reported among this group.

## Competing interests

The author(s) declare that they have no competing interests.

## Authors' contributions

AR was involved in obtaining ethics approval from the appropriate bodies, design of the study, data collection, performing statistical analysis, conducting a detailed literature review and drafting the manuscript. LD conceived of the study, participated in its design, and provided detailed structural comment on, and assistance with drafting the manuscript. JC assisted with the study design and provided comment on the content of the manuscript. All authors read and approved the final manuscript.

## Pre-publication history

The pre-publication history for this paper can be accessed here:


